# A new genus and species of Brontinae from Borneo (Coleoptera, Silvanidae)

**DOI:** 10.3897/zookeys.805.28757

**Published:** 2018-12-11

**Authors:** Takahiro Yoshida, Toshiya Hirowatari

**Affiliations:** 1 Institute of Biological Control, Faculty of Agriculture, Kyushu University, Fukuoka, 819-0395, Japan Kyushu University Fukuoka Japan; 2 Entomological Laboratory, Faculty of Agriculture, Kyushu University, Fukuoka, 819-0395, Japan Kyushu University Fukuoka Japan

**Keywords:** *
Borneophanus
*, Cucujoidea, digitiform sensilla, Malaysia, Telephanini

## Abstract

A new silvanid genus *Borneophanus***gen. n.** is described based on specimens collected from Malaysian Borneo. A new species, *B.spinosus***sp. n.**, is described herein. Digitiform sensilla on the apical maxillary palpomere is reported in Silvanidae for the first time.

## Introduction

The family Silvanidae Kirby, 1837 (Coleoptera, Cucujoidea) includes two subfamilies, Brontinae Blanchard, 1845 and Silvaninae Kirby, 1837, and 62 extant described genera (Thomas and Nearns 2008; Thomas and Leschen 2010; Thomas 2011; Karner et al. 2015; Yoshida et al. 2017). Thomas and Leschen (2010) stated that there are 58 genera in Silvanidae, but they may not have included *Australophanus* Thomas, 2008 described in Thomas and Nearns (2008). In the Brontinae, 12 new genera have been described since the beginning of the 21^st^ Century, and the subfamily contains 23 genera (Thomas 2004, 2011; Thomas and Nearns 2008; Karner et al. 2015). Thomas and Nearns (2008) provided diagnostic character states of the brontine tribe Telephanini LeConte, 1861 and a key to the genera of this tribe. In addition, Karner et al. (2015) described a new telephanine genus *Bolianus* Karner, Salvato & Uliana, 2015 and added the genus to the key of genera by Thomas and Nearns (2008). According to Thomas and Nearns (2008) and Karner et al. (2015), the telephanine genera can be identified by the absence or presence and number of frontal grooves; the presence or absence of the frontoclypeal suture; the shape of antennae; the shape of tarsomere III; the shape of the terminal labial palpomere and/or maxillary palpomere; and the presence or absence of a scutellary striole. The genus *Telephanus* Erichson, 1846 is the most species-rich genus among Silvanidae and is distributed in the New World, Madagascar, and the Mascarene Islands (Thomas 1992; Thomas and Leschen 2010). Although some *Telephanus* species of Madagascar are known to have a scutellary striole, the remaining species of the genus lack it (Thomas 1992; Thomas and Nearns 2008). According to the molecular phylogenetic study of McElrath et al. (2015), the Malagasy *Telephanus* are not sister to New World *Telephanus* species but to the genus *Psammoecus* Latreille, 1829 and likely represent a distinct group. Additionally, Karner (2014) stated that *Psammoecus* is likely composed of several distinct species groups and predicted a further subdivision of this genus. As stated above, among Telephanini, generic and subgeneric classification involves many problems, thus, further taxonomic studies are still needed for the tribe Telephanini. In this paper, we describe a new telephanine genus, represented by a single species, *Borneophanusspinosus* Yoshida & Hirowatari, gen. et sp. n., from Malaysian Borneo.

## Materials and methods

Observations of external characters and dissections were performed under a stereomicroscope (Olympus SZX10). The dissections were made according to the methods of Yoshida and Hirowatari (2014). After observation, the dissected parts were mounted in Euparal on cover glasses, each glued to a piece of cardboard and pinned with the specimens.

Photographs were taken with a digital camera (Canon EOS 7D) and a macro lens (Canon MP-E 65 mm), and composite images were produced using stacking software (Combine ZM). The scanning electron microscopy (SEM) images were obtained using a Hitachi S-3000N.

Depository of the holotype is in the Ehime University Museum, Matsuyama, Japan (**EUMJ**); paratypes are deposited there and in the Australian National Insect Collection, CSIRO, Canberra (**ANIC**).

Technical terms follow Williams (1938), Halstead (1980), Thomas and Leschen (2010) and Yoshida and Hirowatari (2016). Abbreviations and measurements are as follows:

**BL**HL + PL + EL.

**EL** length of elytra measured along the median line.

**EW** greatest combined width of elytra

**HL** length from anterior margin of clypeus to imaginary line between posterior margins of temples in dorsal view measured along the median line.

**HW** greatest width of head across eyes.

**IE** narrowest width of interspace between eyes.

**PL** length of pronotum measured along the median line.

**PW** greatest width of pronotum, excluding teeth.

## Taxonomy

### Borneophanus gen. n.

Taxon classificationAnimaliaColeopteraSilvanidae

http://zoobank.org/E5B80A06-C636-4CFF-AA37-A3A86EB7EB49

#### Type species.

*Borneophanusspinosus* Yoshida & Hirowatari, sp. n.

#### Diagnosis.

Among telephanine genera, this new genus shares the following character states with *Telephanus*, *Psammoecus*, and *Indophanus* Pal, 1982: apical maxillary palpomere securiform; apical labial palpomere securiform; scutellary striole absent (some species of Malagasy *Telephanus* have a scutellary striole). This new genus differs from these genera by the combination of the following character states: distinct pair of longitudinal frontal lines present (absent in *Telephanus*); scutellar shield with a transverse carina and excavate posteriorly (flat in *Psammoecus*); antennomere IV normal (markedly long, approximately twice as long as combined length of II and III in *Indophanus*) (Thomas and Nearns 2008; Sen Gupta and Pal 1996). In addition, this new genus possesses the following characteristic morphology: the asymmetric shaped antennomere X (somewhat asymmetric in *Bolianus*); the sharply protruding elytral apices; the non-folded internal sac; the partly coiled flagellum; and the very long and rolled up spermathecal duct.

#### Description.

**Figure 1.**
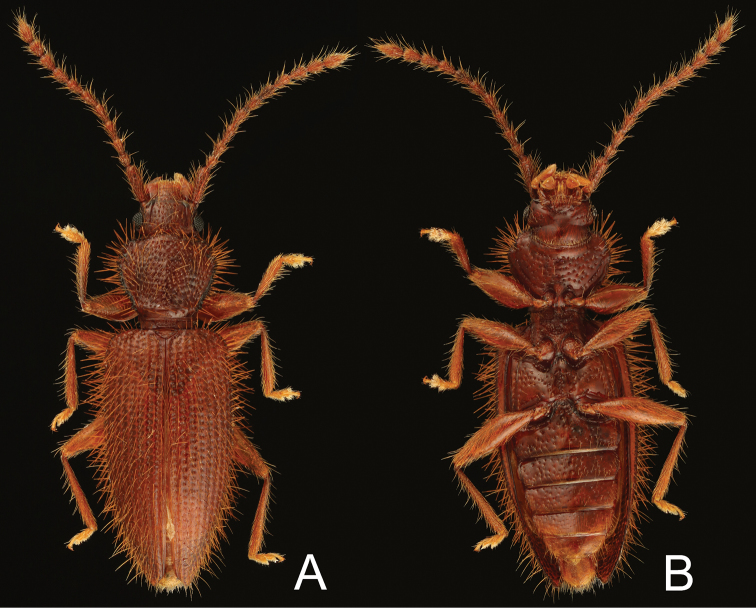
Habitus of *Borneophanusspinosus* gen. et sp. n., holotype, male. **A** dorsal **B** ventral aspect.

**Figure 2.**
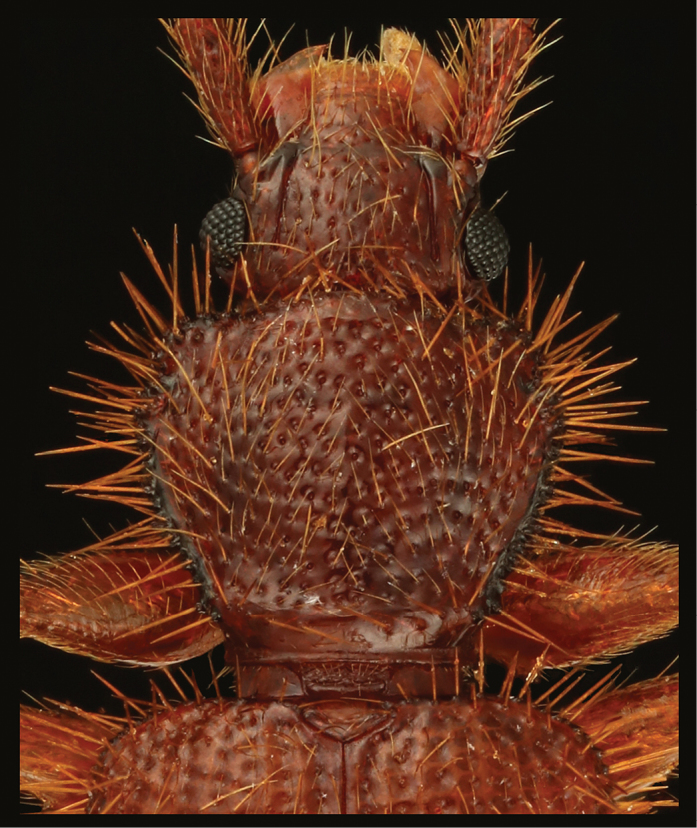
Close-up image of head, pronotum, scutellar shield and basis of elytra of *Borneophanusspinosus* gen. et sp. n., holotype, male.

**Figure 3.**
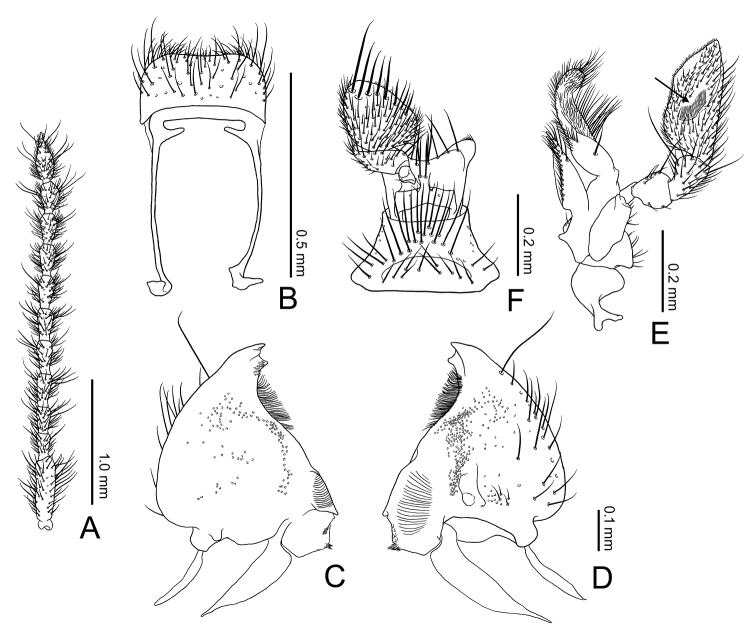
Antenna and mouth parts of *Borneophanusspinosus* gen. et sp. n., paratype, male. **A** Right antenna **B** labrum, dorsal view **C, D** right mandible, ventral view (**C**) and dorsal view (**D**) **E** right maxilla, dorsal view **F** labium, ventral view. An arrow indicates digitiform sensilla.

**Figure 4.**
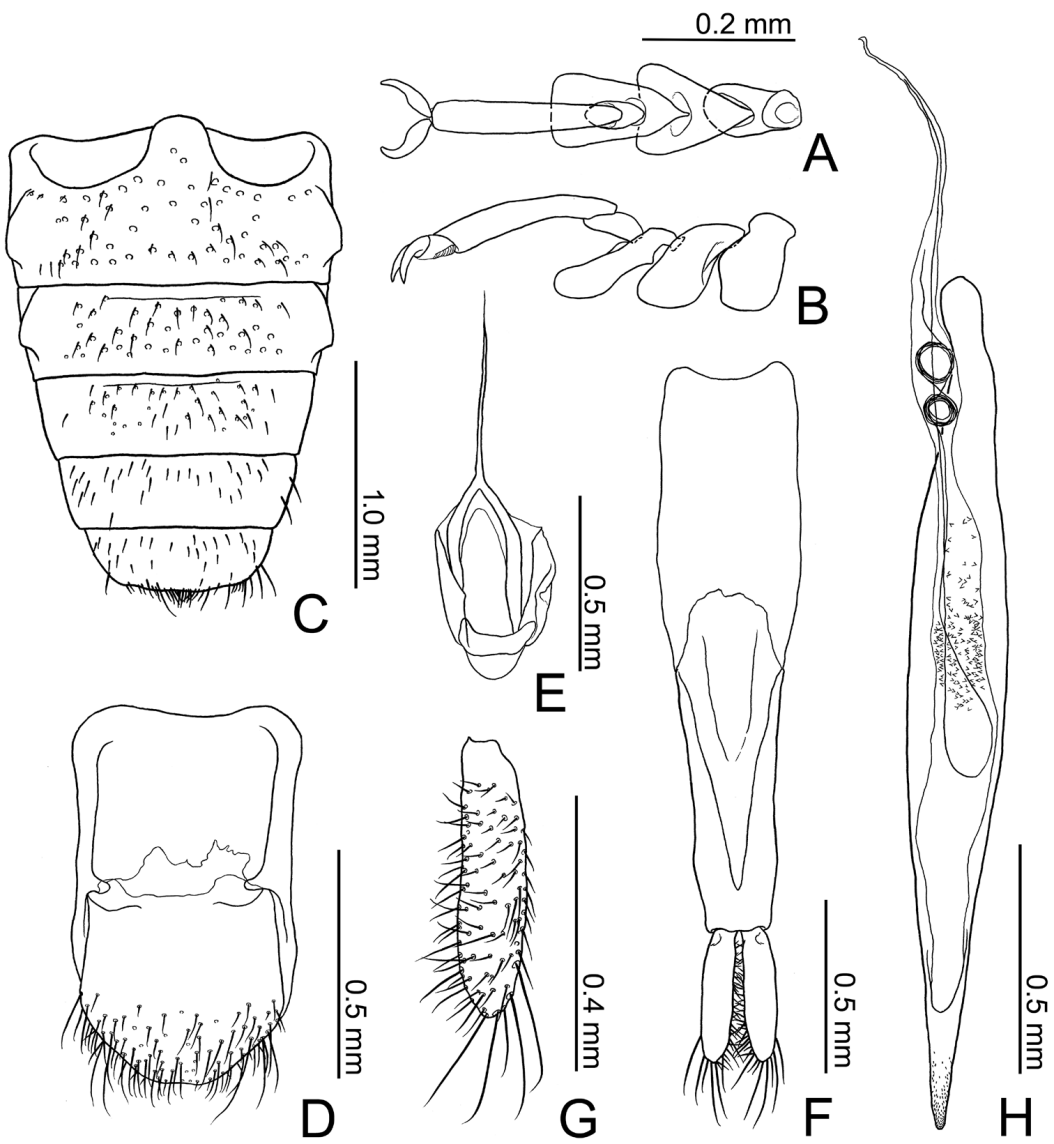
Tarsomeres and abdominal parts including the male genital structures of *Borneophanusspinosus* gen. et sp. n., holotype. **A, B** Tarsomeres, dorsal view (**A**) and lateral view (**B**) **C** abdominal ventries **D** eighth abdominal segments, ventral view **E** spiculum gastrale, ventral view **F** tegmen, ventral view **G** paramere, dorsal view **H** median lobe and internal sac, ventral view.

Body densely covered with pubescence. Head (Figs 1–3) with indistinct frontoclypeal suture, with distinct pair of longitudinal frontal lines; eyes somewhat small, hemispherical; temples moderate in size; labrum (Fig. 3B) rectangular; antenna (Fig. 3A) long, with long scape; antennomere X asymmetrically enlarged; mandible (Fig. 3C, D) tridentate, with a ventral tooth and an inner lateral tooth near apex, ventrally with asperities near the inner tooth, with fine, random punctation, dorsolaterally with medium length to long setae, dorsally with a mycangium near base; maxilla (Fig. 3E) with lacinia and galea; galea divided into distigalea and basigalea; maxillary palp 4-segmented; apical palpomere securiform, dorsally and largely with digitiform sensilla on middle of apical palpomere; labium (Fig. 3F) divided from ligula; prementum widened distally; articulation of palps tight; labial palp 3-segmented; palpomere 2 extended outwards strongly and broadly, with large distal area; palpomere 3 securiform, strongly widened distally; mentum widened proximally, widest near base. Pronotum (Figs 1A, 2) without teeth on lateral margins, with rounded anterior angles, densely covered with setae of various lengths, without microsculpture. Thoracic ventrites (Fig. 1B) with narrowly separated pro- and mesocoxal cavities, with somewhat widely separated metacoxal cavities. Scutellar shield (Figs 1A, 2) with a transverse carina, excavate posteriorly. Legs (Figs 1, 4A, B) somewhat thin; tarsomeres 2 and 3 lobed, not bilobed. Abdominal ventrites (Fig. 4A) with somewhat wide intercoxal process, without sexual dimorphism. Elytra (Figs 1A, 2) long, without scutellary striole; lateral margins very narrowly explanate; apices somewhat elongate and narrowly rounded, with short setae on interstices. Male tergite and sternite VIII square and not divided (Fig. 4D); spiculum gastrale (Fig. 4E) thin and Y-shaped, with branches covered with membrane; median lobe (Fig. 4H) without setae; internal sac (Fig. 4H) not folded, with partly coiled flagellum, exposed around apex of median lobe; parameres (Fig. 4F, G) flat. Female with elongate gonostyli (Fig. 6B); gonocoxite (Fig. 6B) with setae of various lengths; spermathecal duct (Figs 6A, 7A) connected to basal portion of bursa copulatrix, very long, rolled up and forming large ellipsoid.

**Figure 5.**
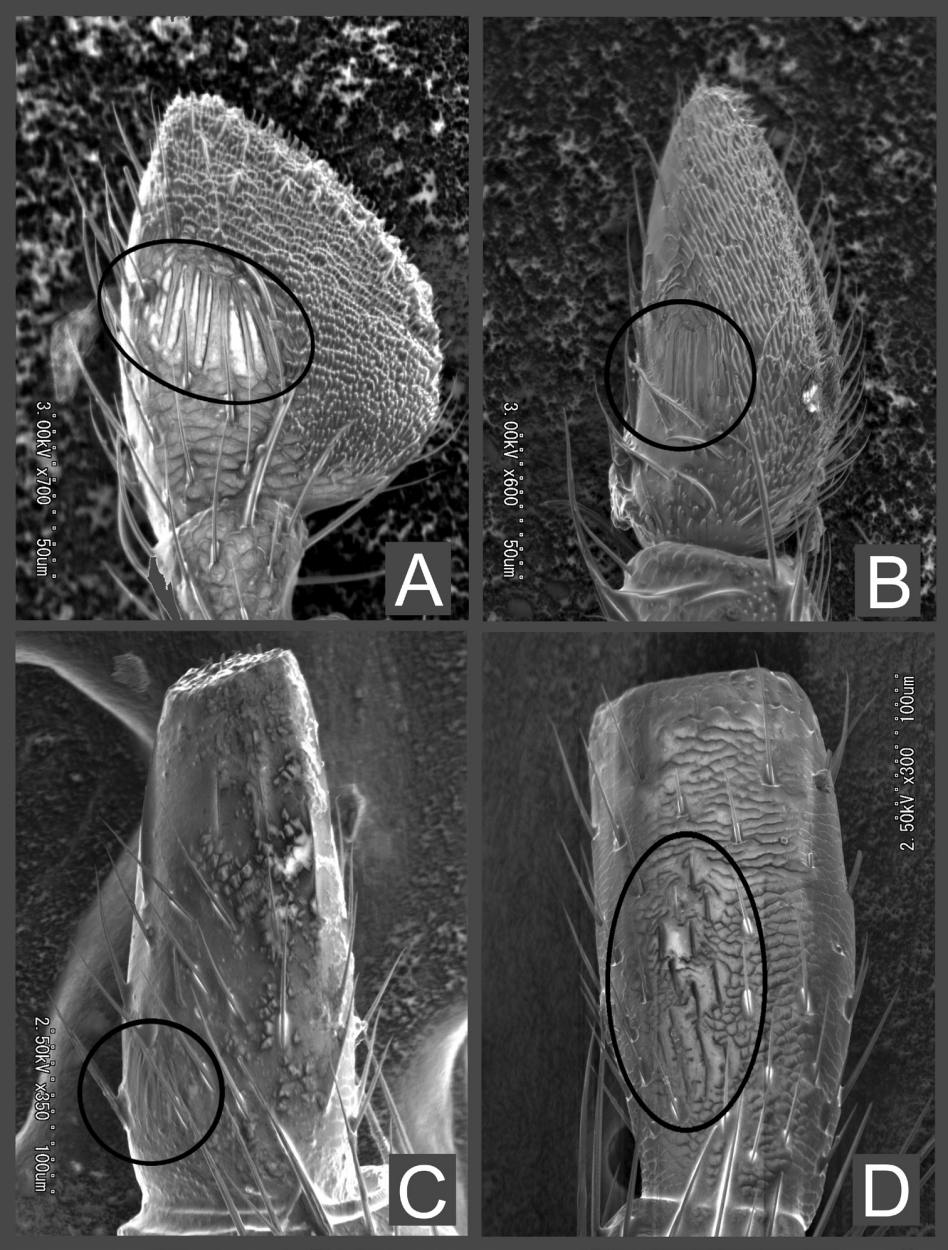
SEM images of apical palpomere of left maxillae (**A–C**) and a right maxilla (**D**), dorsal view, with digitiform sensilla. **A***Psammoecusdentatus***B***Telephanusparadoxus***C***Australohyliotamcleayi***D***Cucujusbicolor*. Circles indicate digitiform sensilla.

**Figure 6.**
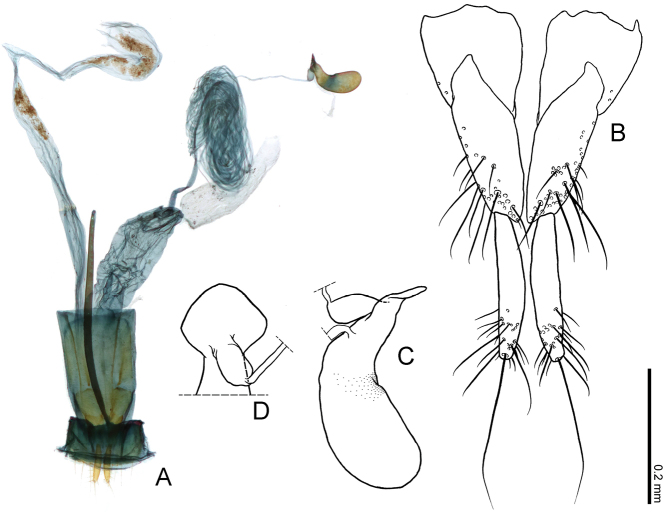
Female genital structures of *Borneophanusspinosus* gen. et sp. n. **A** female genital structures **B** gonocoxites and gonostyli **C, D** spermatheca, lateral view (**C**) and basal view (**D**). Scale bar provided for **B–D**.

**Figure 7.**
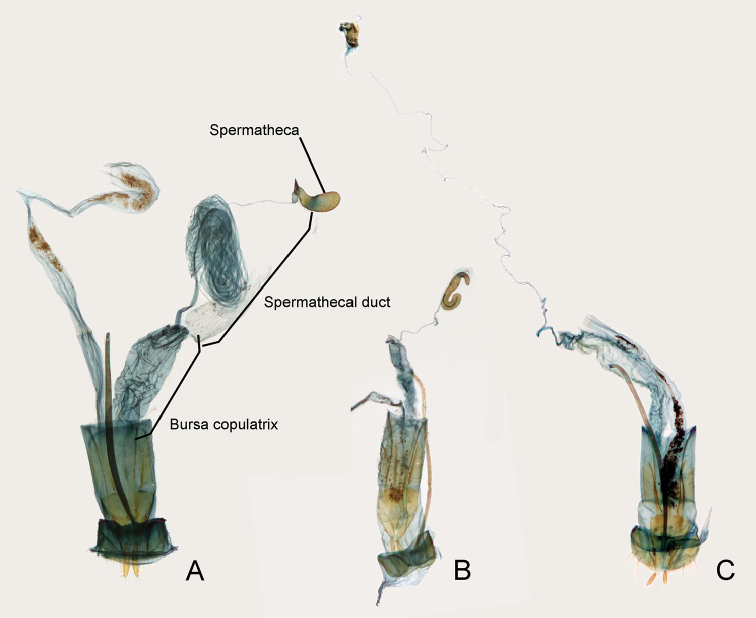
Female genital structures of telephanine species. **A***Borneophanusspinosus* gen. et sp. n. **B***Psammoecusdentatus***C***Cryptamorphadesjardinsi*.

#### Distribution.

Malaysia (Sabah and Sarawak states).

#### Etymology.

The new genus name is composed of two words, the locality, Borneo where this new genus was collected, and the Greek *phanos* meaning bright.

#### Remarks.

We found sensilla, called digitiform sensilla, each located in a groove on the dorsum of the apical maxillary palpomere. Lawrence and Ślipiński (2013) stated that such sensilla on apical maxillary palpomere may be present in adephagan and polyphagan beetles, but they have never been described in Silvanidae. We confirmed these in genera possessing securiform apical maxillary palpomeres (Telephanini: *Psammoecusdentatus* Grouvelle, 1883; *Telephanusparadoxus* Reitter, 1874; *Cryptamorphadesjardinsi* (Guérin-Méneville, 1844), Brontini: *Australohyliotamcleayi* (Olliff, 1885), Cucujidae: *Cucujusbicolor* Smith, 1851), although, these sensilla vary in the position and size of the area among the genera (Fig. 5).

The spermathecal duct of this new genus is extremely long, likely correlated to the partly coiled flagellum of the male genitalia. However, among Telephanini, female genital structures have not been studied. For comparison, we examined the female genitalia of *P.dentatus* (Fig. 7B) and *C.desjardinsi* (Fig. 7C) and found that their spermathecal ducts are apparently shorter than in the new genus. Additionally, we found further differences in the female genitalia (e.g., gonostyli; spermatheca), thus, future comprehensive study of telephanine female genitalia seems likely to provide some useful taxonomic characters and/or their phylogenetic insights.

The biology of the new genus is unknown except for the information that one paratype of the type series was collected by beating foliage. This new genus possesses lobed tarsomeres which are shared by most telephanines and seem to be related to the ecology living on dead leaves (Thomas 1984), thus, they may have ordinary habits of telephanine beetles.

### Borneophanus spinosus sp. n.

Taxon classificationAnimaliaColeopteraSilvanidae

http://zoobank.org/D8D16E65-0970-49BE-BC6E-DB0A4D1A4805

Figs 1 , 2 , 3 , 4 , 6 , 7A

#### Type series.

**Holotype**: male, Poling near Ranau, Sabah State, Malaysia, 26 Apr 1980, M & A Sakai leg. (EUMJ). **Paratypes**: [Sabah State] 1 male and 1 female, same data as the holotype (EUMJ); 1 female, Sandakan Bay (SW), Sapagaya Lumber Camp (2–20 m), 1 Nov 1957, JL Gressitt leg. (ANIC); 1 female, Poring Hot Springs, Mt. Kinabalu National Park (486 m), 8–14 May 1987, DE Bright leg., Beating foliage. (ANIC; Loan from USNMNH 2031682). [Sarawak State] 1 male, Gunong Matang (120 m), 13 Sept 1958, JL Gressitt leg. (ANIC).

#### Diagnosis.

This new species is superficially similar to some *Telephanus* species bearing long spines on lateral pronotum and elytra. This new species can be easily distinguished from these species by the distinct pair of longitudinal frontal lines, the asymmetric shaped antennomere X, and the sharply protruding elytral apices.

#### Description.

**BL**: 4.47–5.28 mm (n = 5). *Coloration* (Fig. 1). Surface yellowish to reddish brown, mostly unicolored; legs and setae lighter colored. *Head* (Figs 1–3) subquadrate, HL 0.61–0.70 mm; HW 0.87–1.03 mm; HW/HL 1.37–1.46; IE/HL 0.90–1.04 (n = 5). Eye roundly protruding, as long as length of antennomere IX. Punctation strong and distinct, absent laterally to frontal lines; ventral surface with sparser punctation, impunctate medially. Pubescence fine, composed of short to long setae, setae located on posterior margin of each puncture or on very minute tubercles lateral to frontal lines. Labrum (Fig. 3B) ventrally with many setae along anterior margin, dorsally with somewhat dense pubescence composed of short to medium length setae. Antenna (Fig. 3A) with long and somewhat thick scape, three times as long as antennomere II; very densely covered with fine and medium length to long setae; antennal length and approximate ratios of antennomere lengths of holotype as follows: 3.10 mm; 3.0 : 1.0 : 1.3 : 1.4 : 1.4 : 1.4 : 1.4 : 1.4 : 1.4 : 1.3 : 2.2. Mandible (Fig. 3C, D) ventrally with a stout tooth protruding apically, with a wide tooth on inner margin near apex, ventrally densely covered with many asperities near the inner tooth, densely, finely pubescent on approx. anterior 1/3 of inner margin, punctation very fine and partly very dense, sparse or absent near apex, lateral and molar regions, dorsally with one long seta near apex and some setae of medium length on outer lateral region, with some short setae near mycangium; mola widely extended posteriorly, with some teeth and dense cuticular spines on posterior 1/3 of inner margin; mycangium located on middle of posterior area, opening towards outer lateral margin, longitudinally depressed on and around mycangium. Maxilla (Fig. 3E): lacinia with two long apical blunt teeth, dorsally with some setae in a longitudinal row along inner margin, few apically, these setae long, with several long setae along apical 2/5 of lateral margin; galea long and flattened, somewhat membranous; distigalea with short to very long, dense pubescence, dorsally with a short row of several short setae and one long seta on posterior half, connected to basigalea by a membrane; palpifer longitudinally oblong, ventrally with several short setae; palpomere 1 very small, extended at inner distal portion; palpomere 2 approx. twice as long as palpomere 1, strongly widened distally, with some short setae, with one very long seta on inner distal portion; palpomere 3 longer than palpomere 2, widened distally, covered with setae of various length, with one very long seta on inner distal portion; palpomere 4 securiform, strongly expanded, 2.5 times as long as palpomere 3, densely covered with short to medium length setae, densely with very short setae along distal margin; stipes ventrally with some medium length setae. Labium (Fig. 3F) distinctly divided from ligula; prementum gradually widened distally, with several long setae along distal margin, with pair of long setae and few short setae near palps; palpomere 1 very small, with few short setae, with a puncture on inner side; palpomere 2 densely covered with many setae of various lengths; palpomere 3 densely covered with many setae of medium length, ventrally with several thick and long to medium length setae near distal margin, distally and densely covered with minute setae; mentum somewhat strongly widening proximally, partly somewhat densely covered with long setae. *Thorax and abdomen* (Figs 1, 2, 4A–C). Pronotum wider than long, PL 1.00–1.18 mm; PW 1.14–1.44 mm; PW/PL 1.15–1.24 (n = 6), enlarged around anterior angles and anterior 1/3 of lateral margins, with numerous minute setiferous tubercles densely covering lateral margins and sparser on anterior and posterior margins, with a long spine on each tubercle, somewhat densely covered with fine and long pubescence except on margins; punctation coarse, similar to vertex. Thoracic ventrites with punctation sparser than on dorsum, with pubescence shorter and sparser than on dorsum, densely covered with numerous short setae on anterior margin of proventrite; intercoxal process of procoxae widest at posterior 2/5, strongly narrowed around anterior 1/5 to 2/5, somewhat extended around posterior angles; mesocoxal process narrowed posteriorly, widened around apex. Scutellar shield (Figs 1A, 2) approx. twice as wide as long, wider than eye length, with few short setae. Legs somewhat thin; trochanters small, with sparse setae of various lengths, with one long seta; femora somewhat thin, densely covered with thin and medium length setae; profemora a little expanded; tibiae thin but gradually widening distally, with similar pubescence of femora, with some conical setae around apices; tarsomere 5 long, approx. 3 times as long as 4 (Fig. 4A,B). Abdomen (Fig. 4C) 1.5 times as long as wide; intercoxal process somewhat wide and moderately rounded anteriorly; 1^st^ and 2^nd^ abdominal ventrites with strong lateral protuberances; punctation sparse, weaker toward posterior; setae similar to those on thoracic ventrites. *Elytra* (Figs 1A, 2) long, sub-parallel for approximately the anterior 2/3, EL 2.84–3.40 mm (n = 6); EW 1.46–1.76 mm at approximately 1/3 length (n = 6); EW/EL 0.51–0.52 (n = 6); EW/BL 0.32–0.34 (n = 5); apices sharply protruding and triangular, with rows of punctures almost as wide as interstices, with short and thin setae on anterior margins of the punctures, with very minute setiferous tubercles on the spaces between rows, with long and thin setae on the tubercles, these setae near lateral margins thick, densely with many spines of long and medium lengths on lateral margins. *Male genitalia* (Fig. 4D–H). Tergite VIII (Fig. 4D) square, longer than wide, with rounded posterior margin, with many setae of various lengths around posterior margin; lateral margins protruding inwards near middle and connected with sternite VIII; sternite VIII (Fig. 4D) square, almost as long as wide, with rounded posterior margin, with many setae of various lengths on posterior half, with few long setae along posterior margin near posterior angles; spiculum gastrale (Fig. 4E) Y-shaped, with thin strut, bifurcating at around midlength, with simple branches; branches covered with membrane. Median lobe (Fig. 4H) very long and thin, gradually narrowed toward apex, with internal sac exposed for the apical 1/6, densely punctate around apex. Parameres (Fig. 4F, G) long, flattened, subparallel, dorsally excavated near bases, dorsally without punctures and setae, ventrally densely covered with many setae of various lengths except on bases, with a few long setae along apical margins. Phallobase (Fig. 4F) long and subparallel; tegminal strut connected to basal piece around midlength; basal piece long; posterior margin of upper layer very deeply and sharply incised; posterior margin of lower layer widely extended anteriorly, somewhat membranous. Internal sac (Fig. 3H) minutely spinous around midlength; flagellum long, with two coiled sections near posterior 1/3.

**Female genitalia** (Figs 6, 7A). Gonostyli (Fig. 6B) with a long seta and setae of various length on apical 1/3, shorter than gonocoxite; spermatheca (Fig. 6C, D) bean-shaped and gradually enlarged distally, basally with a small sac connected to spermathecal duct, basis somewhat strongly explanate, with membranous spermathecal gland on basal 1/3, somewhat membranous near midlength.

#### Distribution.

Malaysia (Sabah and Sarawak states).

#### Etymology.

The specific name means thorny and indicates the characteristic long spines.

#### Remarks.

This new species possesses characteristic long spines covering the lateral margins of pronotum and elytra. In possessing such setation, some *Telephanus* species (e.g., *T.paradoxus* and *T.sellatus* Sharp, 1899) are superficially similar to this new species. They can be easily distinguished by the diagnostic character states of these genera. Otherwise, like *Telephanus*, such setation often occurs as a taxonomic character at the species or species group level; thus, the setal situation is not regarded as a character of this new genus.

## Supplementary Material

XML Treatment for Borneophanus

XML Treatment for Borneophanus spinosus
